# Test-retest reliability of Common Mental Disorders Questionnaire (CMDQ) in patients with total hip replacement (THR)

**DOI:** 10.1186/s40359-014-0032-5

**Published:** 2014-09-08

**Authors:** Randi Bilberg, Birgitte Nørgaard, Kirsten Kaya Roessler, Søren Overgaard

**Affiliations:** Department of Orthopaedic Surgery, Kolding Hospital, a part of Lillebaelt Hospital, Odense, Denmark; Institute of Psychology, University of Southern Denmark, Campusvej 55, 5230 Odense, Denmark; Emergency Department, Kolding Hospital, a part of Lillebaelt Hospital, Odense, Denmark; Department of Orthopaedics and Traumatology, Odense University Hospital, Odense, Denmark; Institute of Clinical Research, University of Southern Denmark, Odense, Denmark

**Keywords:** Test-retest, Reliability, Mental disorders, CMDQ, Kappa, Missing

## Abstract

**Background:**

The Common Mental Disorders Questionnaire (CMDQ) is used to assess patients’ mental health. It has previously been shown to provide a sensitive and specific instrument for general practitioner setting but has so far not been tested in hospital setting or for changes over time (test-retest). The aim of this study is, by means of a test-retest method, to investigate the reliability of the instrument over time with total hip replacement (THR) patients.

**Methods:**

Forty-nine hip osteoarthritis patients who had undergone THR answered the questionnaire twelve months after their operation. Fourteen days later they completed it again. Covering emotional disorder, anxiety, depression, concern, somatoform disorder and alcohol abuse, the questionnaire consists of 38 items with six subscales, each of which has between 4 to 12 items. A five-point Likert scale (from 0–4) is used.

**Results:**

For each of the 38 questions, a quadratic-weighted Kappa coefficient of 0.42 (0.68 – 0.16) to 0.98 (1.00 – 0.70) was found. A Cronbach’s alpha of 0.94 for all the questions indicated high internal consistency.

**Conclusion:**

The results showed a moderate to almost perfect reliability of CMDQ of this specific population.

**Trial registration:**

Current Controlled Trials: NCT01205295

## Background

A review of the literature shows a generally increasing interest in the influence of mental disorders in patient’s experience of pain (Linton, 2000; Linton, 2005), but in orthopaedic and other departments responsible for surgical procedures, the focus remains centred on physical functions (in relation to indication for surgery) (Okoro et al. 2012; Sedrakyan et al., 2011; Veenhof et al. 2012). A small number of studies, e.g. of hip-operated patients, have shown an association between mental disorder and outcomes of surgery, but further research using a more sensitive and specific questionnaire is still called for (Rolfson et al. 2009; Hossain et al., 2011; Dawson et al., 2001). Annually, approximately 10, 000 patients undergo total hip replacement (THR) in Danish hospitals. About 20 percent of the patients experience pain postoperatively and some of them even worse pain then preoperatively; which indicates the need for the evaluation of predictors for pain development (Judge et al. 2010). A positive correlation between patients’ pain and their mental health is well established (Linton, 2005), which prompted a 2012 systematic review to request further investigation of the effect of psychological factors in THR patients (Vissers et al., 2012).

The existing studies of psychological factors in THR patients have investigated anxiety and depression (Vissers et al., 2012), but so far there has been a little interest in patients’ levels of concern as part of their mental health. The CMDQ provides a tool for assessing patients’ mental health focusing on concern, anxiety, depression, somatoform disorders and alcohol abuse (Sogaard, 2009) and was developed by Christensen and Fink at Aarhus University in 2004 to use in primary care. The definition of mental disorders is somatisation, anxiety, depression, concern and alcohol abuse (Christensen et al., 2005b).

The questionnaire has previously been used for assessment of the mental health status of various groups, such as medical patients, neurological patients and patients in general practises (Fink et al. 2004; Christensen et al., 2005a). A study from 2009 investigated long-term sickness absence (Sogaard & Bech, 2009), but this is the first study to investigate the instrument’s reliability in relation to (changes over) time (in a test-retest format) in a hospital setting, although Mokken analysis was used (in 2010) to assess responsiveness and standardised response mean of CMDQ in primary care patients (Christensen et al. 2010).

The present study aims is to investigating the reliability of CMDQ by means of a test-retest method in patients who have undergone THR.

## Methods

### The questionnaire

The 38-items questionnaire was developed in 2003 with the aim of supporting general practitioners in their assessment of the patients’ mental health. It has six subscales: SCL-SOM, Whiteley-7, SCL-ANX4, SCL-8, SCL-DEF6 and CAGE (Christensen et al., 2005a). A Danish translation was made in a two-stage process and then validated using the Schedules for Clinical Assessment in Neuropsychiatry (SCAN) interview as a golden standard (κ = 0.86) (Christensen et al., 2005a; Christensen et al., 2005b; Christensen et al., 2003).

### SCL-R-90 subscales

Four of the subscales, SCL-SOM, SCL-ANX4, SCL-8 and SCL-DEF6, are based on the Symptom Checklist-90-revised (SCL-R-90), as developed and validated by Derogatis et al. in 1973 (Derogatis et al. 1973). Numerous studies have since demonstrated it’s validated and reliability (Holi et al. 1998; Schmitz et al., 2000; Olsen et al. 2004).

The 12-item SCL-SOM subscale assesses is somatic distress (1–12) (item numbers shown in Table 1). The subscale SCL-ANX4 has 4 items (21–24) measuring anxiety. Emotional disorders are assessed in the 7-itme SCL-8 subscale (22–29), while the SCl-DEF6, with 6 items (28–33), is a depression measure.

**Table 1:** **Weighted quadratic Kappa with confidence intervals (IC) and Cronbach’s Alpha by questions**

**During the last 4 weeks how much were you bothered by:**	**Kappa (IC)**	**Kappa (IC). highest value (four) instead of missing**	**Kappa (IC). smallest value (zero) instead of missing**	**Kappa (IC). Mean of individual score of the questions instead of missing**	**Cronbach’s alpha (n = 49)**
1. Headaches?	0.67 (0.95 – 0.39)	0.31 (0.57 – 0.03)	0.67 (0.94 – 0.40)	0.52 (0.79 – 0.25)	0.9409
2. Dizziness or faintness?	0.80 (1.00- 0.52)	0.25 (0.52 – -0.02)	0.80 (1.00 – 0.53)	0.80 (1.00 – 0.53)	0.9425
3. Pains in the heart or chest?	0.42 (0.68 – 0.16)	0.22 (0.49 – -0.05)	0.41 (0.16 – 0.66)	0.48 (0.75 – 0.21)	0.9386
4. Pains in the lower back?	0.61 (0.89 – 0.33)	0.52 (0.79 – 0.25)	0.62 (0.89 – 0.35)	0.61 (0.88 – 0.34)	0.9427
5. Nausea or upset in the stomach?	0.80 (1.00 – 0.52)	0.62 (0.89 – 0.35)	0.80 (1.00 – 0.53)	0.81 (1.00 – 0.54)	0.9412
6. Soreness of your muscles?	0.69 (0.97 – 0.41)	0.71 (0.98 – 0.43)	0.60 (0.87 – 0.33)	0.67 (0.94 – 0.40)	0.9395
7. Trouble getting your breath?	0.77 (1.00 – 0.52)	0.54 (0.76 – 0.32)	0.77 (1.00 – 0.52)	0.82 (1.00 – 0.28)	0.9394
8. Hot or cold spells?	0.69 (0.97 – 0.41)	0.69 (0.96 – 0.42)	0.69 (0.96 – 0.42)	0.69 (0.96 – 0.42)	0.9386
9. Numbness or tingling in parts of your body?	0.54 (0.78 – 0.30)	0.40 (0.62 – 0.18)	0.54 (0.77 - 0.31)	0.63 (0.90 – 0.36)	0.9412
10. A lump in your throat?	0.55 (0.82 – 0.30)	0.42 (0.66 – 0.16)	0.22 (0.47 – -0.03)	0.67 (0.94 – 0.40)	0.9388
11. Feeling weak in parts of your body?	0.72 (0.99 – 0.45)	0.50 (0.75 – 0.25)	0.69 (0.94 – 0.44)	0.71 (0.98 – 0.44)	0.9392
12. Heavy feelings in your arms or legs?	0.68 (0.95 – 0.41)	0.57 (0.82 – 0.32)	0.68 (0.93 – 0.43)	0.63 (0.90 – 0.36)	0.9389
13. Worries that there is something seriously wrong with your body?	0.72 (1.00 – 0.44)	0.52 (0.79 – 0.25)	0.72 (0.99 – 0.45)	0.69 (0.96 – 0.42)	0.9375
14. Worries that you suffer a disease you have read or heard about?	0.54 (0.82 – 0.26)	0.38 (0.65 – 0.11)	0.54 (0.81 – 0.27)	0.52 (0.79 – 0.25)	0.9402
15. Many different pains or aches?	0.60 (0.88 – 0.32)	0.47 (0.74 – 0.20)	0.60 (0.87 – 0.33)	0.45 (0.18 – 0.72)	0.9379
16. Worries about the possibility of having a serious illness?	0.71 (0.98 – 0.42)	0.51 (0.78 – 0.24)	0.71 (0.97 – 0.43)	0.67 (0.94 – 0.40)	0.9404
17. Many different symptoms?	0.64 (0.90 – 0.38)	0.38 (0.65 – 0.11)	0.62 (0.89 – 0.37)	0.61 (0.88 – 0.34)	0.9396
18. Thoughts that the doctor may be wrong if telling you not to worry?	0.58 (0.86 – 0.30)	0.35 (0.62 – 0.08)	0.58 (0.85 – 0.31)	0.58 (0.85 – 0.31)	0.9404
19. Worries about your health?	0.69 (0.97 – 0.41)	0.47 (0.74 – 0.20)	0.69 (0.96 – 0.42)	0.66 (0.93 – 0.39)	0.9389
20. Recurrent thoughts about you having an illness that you have trouble getting out of you head?	0.64 (0.90 – 0.38)	0.43 (0.70 – 0.16)	0.64 (0.91 – 0.37)	0.65 (0.92 – 0.38)	0.9399
21. Feeling suddenly scared for no reason?	0.75 (1.00 – 0.47)	0.50 (0.75 – 0.25)	0.75 (1.00 – 0.48)	0.73 (1.00 – 0.46)	0.9393
22. Nervousness or shakiness inside?	0.65 (0.93 – 0.37)	0.65 (0.92 – 0.38)	0.65 (0.92 – 0.38)	0.65 (0.92 – 0.38)	0.9376
23. Spells of terror or panic?	0.73 (1.00 – 0.46)	0.44 (0.71 – 0.17)	0.73 (1.00 – 0.46)	0.76 (1.00 – 0.49)	0.9407
24. That you worry too much?	0.84 (1.00 – 0.56)	0.82 (1.00 – 0.55)	0.78 (1.00 – 0.51)	0.80 (1.00 – 0.55)	0.9401
25. Feeling fearful?	0.67 (0.95 – 0.39)	0.67 (0.94 – 0.40)	0.67 (0.94 – 0.40)	0.67 (0.94 – 0.40)	0.9388
26. Feeling hopeless about the future?	0.84 (1.00 – 0.56)	0.70 (0.97 – 0.43)	0.84 (1.00 – 0.57)	0.84 (1.00 – 0.57)	0.9365
27. Feeling everything is an effort?	0.70 (0.98 – 0.42)	0.70 (0.97 – 0.43)	0.66 (0.93 – 0.39)	0.69 (0.96 – 0.42)	0.9373
28. Feeling blue?	0.73 (1.00 – 0.45)	0.45 (0.72 – 0.18)	0.73 (1.00 – 0.46)	0.72 (0.99 – 0.45)	0.9377
29. Feelings of worthlessness?	0.84 (1.00 – 0.56)	0.68 (0.95 – 0.41)	0.84 (1.00 – 0.57)	0.79 (1.00 – 0.52)	0.9372
30. Thoughts of ending your life?	0.97 (1.00 – 0.69)	0.68 (0.95 – 0.41)	0.97 (1.00 – 0.70)	0.97 (1.00 – 0.70)	0.9383
31. Feeling of being trapped or caught?	0.98 (1.00 – 0.70)	0.65 (0.92 – 0.38)	0.95 (1.00 – 0.68)	0.91 (1.00 – 0.64)	0.9373
32. Feeling lonely?	0.89 (1.00 – 0.60)	0.67 (0.92 – 0.42)	0.81 (1.00 – 0.54)	0.78 (1.00 – 0.51)	0.9376
33. Blaming yourself for things?	0.75 (1.00 – 0.47)	0.45 (0.72 – 0.15)	0.75 (1.00 – 0.48)	0.65 (0.92 – 0.38)	0.9400
**Within the last year, have you ever……**					
34. Felt you ought to cut down on your drinking?	0.89 (1.00 – 0.60)^1^	0.90 (1.00 – 0.63)^1,2^	0.89 (1.00 – 0.62)^1,2^	0.82 (1.00 – 0.59)^1,4^	0.9426
35. Been annoyed by people criticizing your drinking?	1^1^	0.85 (1.00 – 0.58)^1,2^	1^1,2^	0.41 (0.55 – 0.27)^1,4^	
36. Felt bad or guilty about your drinking?	0.79 (1.00 – 0.50) ^1^	0.63 (0.88 – 0.38)^1,2^	0.79 (1.00 – 0.52)^1,2^	0.47 (0.63 – 0.31)^1,4^	0.9415
37. Had a drink in the morning to steady your nerves or get rid of a hangover?	1^1^	0.79 (1.00 – 0.51)^1,2^	1^1,2^	0.39 (0.53 – 0.25)^1,4^	
38. **Overall, would you say your health is:**	0.56 (0.84 – 0.28)	0.53 (0.80 – 0.26)^3^	0.56 (0.83 - 0.29)^3^	0.52 (0.79 – 0.25)^5^	0.9392

The second and third columns in Table 1 show the results of the analysis of weighted quadratic Kappa where the missing values have been changed to either the highest or the smallest possible score values in each question. The fourth column shows the results of changed missing data to individual mean scores.

^1^Analysed by Kappa as the questions require a dichotomous answer.

^2^Highest value is 1 and the smallest 0 (0–1).

^3^Highest value is 5 and the smallest 1 (1–5).

^4^The mean of the all responses to the question instead of the mean of the individual’s mean.

^5^The mean of the question instead of the individual mean as it was one question with the score from one to five.

Missing data and weighted quadratic Kappa (IC) by the questions

### Other subscales

The remaining two subscales in CMDQ are Whiteley-7 (8-items) and CAGE (4-items), which assess illness concern and alcohol abuse respectively in items 13 – 20 and 34 – 37. The Whiteley-7 is based on the 6-items Whiteley index, developed in the 1960s by Pilowsky (1975). It has been translated and validated for use in Danish settings by Fink et al. (2004). The CAGE questionnaire was first cited in 1974 by Mayfield et al. (Mayfield et al. 1974). It has since been translated and validated in several studies (Castells MA FAU et al, 2005; Johnson et al. 2005; Philpot et al., 2003; Knight JR et al. 2003; Saitz et al., 1999; Masur & Monteiro, 1983; Christensen et al., 2005a; Ewing, 1984).

### Response categories in CMDQ

In CMD – SQ, items 1 – 33, patients’ responses were scored on a five-point Likert scale with 0 for “No symptoms at all”, 1 for “A little”, 2 for “Moderately”, 3 for “Quite a bit” and 4 for “Extremely”. The CAGE scale (items 34 – 37) required dichotomised yes/no answers. In the last item, number 38, the patients assessed their own overall health on a five-point Likert scale ranging from “Excellent” (5 points) to “Very good”, “Good”, to “Fair” and “Poor” (1 point) (Sogaard, 2009a; Christensen et al., 2005a)

### Subjects

**Figure 1:**
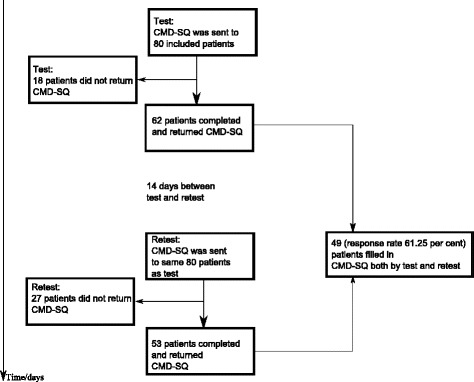
**Flowchart of patients included in test of the reliability of CMD-SQ (Common mental disorders - screening questionnaire).**

A total of 80 hip osteoarthritis patients who underwent a THR 12 months previously were invited to participate in the study. The questionnaires were sent by land mail and had to be completed twice with an interval of 14 days between them (Figure 1). A stamped and addressed envelope was enclosed for returning the completed forms.

A total of 49 patients answered the questionnaire twice (response rate 62%) (Figure 1). There were no significant differences in age and gender between the groups who filled in the questionnaire by test and retest. The final included patients (n = 49) did not significantly differ from non-responders referring to age and sex (n = 31) (Table 2).

**Table 2:** **Tests of age and gender between responders and non-responders**

	**Gender** ^**1**^ **mean (SD)**	**Age mean (SD)**
Responders	0.49 (0.5)	67.7 (9.7)
Non-responders	0.45 (0.5)	70.0 (9.3)
Differences (p-values)	p = .74	p = .30

^1^Men are equal to zero and women are equal to one.

### Ethics statements

The study was presented and approved of The Regional Scientific Ethical Committee for Southern Denmark and the Danish Data Protection Agency (J.nr. 2009-41-3896).

### Statistical analyses

Expect for the four items assessing alcohol abuse (CAGE), all questions were evaluated for test-retest reliability by use of the quadratic weighted Kappa coefficient (Table 1). For the CAGE items, a Kappa coefficient without weighting was used, requiring either a “yes” or a “no” response. According to Landis & Koch, quadratic weighted Kappa coefficients ≤ 0.2 are slight, ≥ 0.2 to 0.4 are fair, while ≥ 0.4 to 0.6 are considered moderate; results ≥ 0.6 to 0.8 are rated as substantial, while ≥ 0.8 to 1.0 as almost perfect (Landis & Koch, 1977).

In order to identify inter-question correlations (internal consistency), we tested all 38 questions in the first test using Cronbach’s alpha coefficient. T-tests were used to analyse for gender and age differences between responders and non-responders. The subscales and the total scores were analysed by paired t-test, quadratic weighted Kappa and Cronbach’s alpha coefficient as to investigate the differences between first and second measurement of the patients.

To detect a possible bias caused by missing responses, the results of the quadratic weighted Kappa were tested in a three-step procedure. In the first step, all missing values were substituted by the lowest possible score (zero), as recommended by Christensen et al. (Christensen et al., 2005a). In the second step, the highest scores for each question were used (Streiner & Norman, 2008). Then, the quadratic weighted Kappa was then calculated by t-test for comparison with the original results of quadratic weighted Kappa test.

A 95% confidence interval was calculated for each test result. All analyses were done using Stata, version 11 (StataCorp. 2001. Statistical Software: Release 11. College Station, TX: Stata Corporation).

## Results

### Weighted quadratic Kappa coefficient analysis the total score and subscales of CMDQ

In Table 3 the results of the total score of the questionnaire and the subscales are shown by a weighted quadratic Kappa from 0.77 with a Standard Error (SE) at 0.16 to 0.90 SE (0.15). The mean score with standard deviation (SD) of every subscale and the total score are also shown in Table 3. The results between first and second measurement showed no-significant differences.

**Table 3:** **Total sum scores first and second measurements; weighted quadratic Kappa and Cronbach’s alpha at the subscales and the total score of CMDQ**

**Subscales (question number)**	**First time Mean (SD)**	**Second time Mean (SD)**	**Difference between first and second mean by paired t-test (p-values)**	**Kappa (SE)**	**Cronbach’s alpha**
DEF-SLC (28–33)	1.7 (3.9)	2.0 (4.4)	p = .58	0.90 (0.15)	0.96
Whitley-7 (13 – 20)	2.3 (3.5)	2.7 (4.4)	p = .06	0.86 (0.14)	0.93
SCL-ANX (21–24)	1.1 (2.1)	1.2 (2.6)	p = .52	0.86 (0.14)	0.92
SCL-8 (22–29)	3.0 (5.1)	3.1 (5.8)	p = .80	0.89 (0.14)	0.94
CAGE (34–37)	0.3 (0.6)	0.3 (0.6)	p = .57	0.90 (0.15)	0.95
SOM-SCL (1–12)	5.0 (6.1)	4.1 (4.2)	p = .17	0.77 (0.16)	0.69
Total	14.7 (12.4)	14.3 (12.1)	p = .48	0.83 (0.17)	0.75

### Weighted quadratic Kappa coefficient analysis for all questions

The results of the weighted quadratic Kappa coefficient for all questions are shown in Table 1. The highest value of Kappa was found for Question 31 (0.98 (CI: 1.0 - 0.70) “During the last 4 weeks how much were you bothered by feeling of being trapped or caught?”); Question 3 had the lowest value, at 0.42 (CI: 0.68 - 0.16) (“During the last 4 weeks how much were you bothered by pains in the heart or chest?”). For Questions 35 and 37, the Kappa coefficient was 1, indicating no differences between test and retest results.

### Cronbach’s alpha analysis

The mean result of the Cronbach’s alpha was 0.9410 for all questions collapsed (Table 1), indicating good internal consistency. No results were obtained for Question 35 and 37, as only one patient answered them in the test while there were no responses in the retest. The two questions required either a “yes” or “no” response. The patient who answered “yes” at test is answering with missing in retest. A Cronbach’s alpha cannot be assess to so small differences in the answering between test and retest from the patients (Vet, 2011).

### Analysis of missing values

The results of the analyses of missing data are shown in Table 1. In general, responders were careful to answer the questions; there were seven missing answer for questions 10 and 36, which has the lowest response frequency. Substituting missing values for zero, a weighted quadratic Kappa coefficient was calculated (mean value 0.71, SD 0.03) and by a t-test compared to a weighted quadratic Kappa coefficient included missing values (mean value is (0.72, SD 0.02), where was no significant (p = 0.060) difference between the Kappa coefficient values. When missing value were substituted by patient’ individual mean scores or by the highest score, the weighted quadratic Kappa coefficients obtained were significantly lower, respectively p = 0.0214 and p < 0.001 than a weighted quadratic Kappa with included missing values.

## Discussion

The aim of this study was to investigate the test-retest reliability of CMDQ. The results of the weighted quadratic Kappa tests showed moderate to almost perfect grade of reliability of questionnaire with reference to Landis and Koch’s classification of Cohen’s Kappa (Landis & Koch, 1977). Originally, the CMDQ was designed with a view to offering a base-line for general practitioners’ discussion of mental health issues with their patients (Christensen et al., 2005b), rather than a tool offering definite results as to whether a patient suffers from e.g. depression. Although Kappa coefficient values as low as 0.42 (Question 3) were obtained, this should not be considered a problem as the CMDQ was never intended to stand alone without any further examination of patients. Some researchers consider all results beyond 0.40 as clinically useful (Sim & Wright, 2005), whereas other regard 0.90 as clinically relevant (Streiner & Norman, 2008). However, the most import is what consequences there will be of the result of the instrument in clinical practice.

The results of the subscales are from 0.83 to 0.90 and consider as clinical relevant. The total score of CMDQ showed a Kappa value at 0.77, but normally it will never be used as a result of a screening at patients, when it gives no mean to measure patients’ depression, anxiety and so on in a total score.

### Study limitations

The questionnaire was sent twice to 80 patients, but only 49 returned both forms. While the Dutch Cosmic Group regards close to 100 participants as the optimum for test-retest studies, it sees 50 participants as acceptable (Vet, 2011). The Dutch Cosmic Group is approximately 50 experts in psychometrics, epidemiology, statistics and clinical medicine who started a international Delphi group with standards and definition of the terminology for the selection of health measurement instruments in 2010 (Vet, 2011). We recommend future test-retest reliability studies to take more than 80 participants into the study from the beginning in relation to the response rate.

A key question is whether the participants’ mental health had changed in the time between the two measurements. This could be controlled by including a global rating question (Vet, 2011) to assess on the respondents self-awareness, we chose not do so.

### Study strengths

The question of the optimum time span between the two measurements in a test-retest format is contentious. Some argue for a 24 – 72 hours interval, while others prefer more than 14 days between the two measurements (Berendes et al. 2010; Frost et al., 1998). A general solution cannot be found as the most suitable interval would depend on the focus of the specific measurement. If that focus is likely to change over short time, the interval should be narrow, but this involves a risk of a recall bias to influence the result, the interval must depend on the focus of the measurement (Fayers & Machin, 2007; Streiner & Norman, 2008). The 14-day interval used for the present study minimizes such a risk as it is difficult to remember the answers for 38 questions over a fortnight.

As the participants of this study had had their THR 12 months before answering the questionnaires, it seemed reasonable to expect the outcome of the operation to be stable (Gogia et al. 1994; Brown et al. 1980); hence we assumed the same to be true for their mental health and thereby we can used the interval of a fortnight between the two measurements.

### Missing values

The present study evaluated missing values in three differences steps in order to identity the best way to handle the problem about missing values in this population using CMDQ. When missing values were replaced by the smallest possible score, zero, the Kappa results showed no significant change. Shrive et al. recommend replacing missing values by the individual mean score (Shrive FM FAU et al. 2006), but as this would entail compromising with a lower mean of the weighted quadratic Kappa coefficient in the reliability of the CMDQ in the specific population. We cannot recommend substituting the individual mean scores for the missing values, if the goal is to have the highest possibly Kappa value.

### Kappa vs. intra correlation coefficient

It has been discussed whether the reliability of the questionnaires with an ordinal scale should be analysed by a weighted Kappa coefficient or by an intra-class correlation coefficient (ICC) (Vet, 2011; Streiner & Norman, 2008). The analyses presented here follow the Dutch COSMIN Group’s recommendation to use a weighted quadratic Kappa coefficient for an ordinal and not normally distributed scale. This has the advantage of allowing our results to be compared to ICC results of similar studies (Vet, 2011). Using a weighted quadratic Kappa assumes equidistant between the response categories (Vet, 2011), something that is not discussed in the literature in CMDQ (Christensen et al., 2005a).

### Cronbach’s alpha

The Cronbach’s alpha assesses the internal consistency of the questionnaire, which reflects the interrelatedness among the items (Mokkink et al., 2010). Often it is the only reported value of the scale (Streiner & Norman, 2008). The reliability of Cronbach’s alpha value must be assessed against other measures of score reliability as its scores are relatively easy to manipulate. The result of the Cronbach’s alpha was 0.94 for all questions collapsed, which is close to the optimal 0.90 (Streiner & Norman, 2008). Cronbach’s alpha is sensitive to the number of the items in the questionnaire and the sample size. With a heterogeneous patient group and many questions, the result of Cronbach’s alpha will increase with the number of questions. In this study, the group was homogeny at age, gender and the focus on the disease. Cronbach’s alpha was an extra analysis of the data and it confirmed the finding of a moderate to almost perfect degree of reliability of CMDQ for patients with THR.

## Conclusion

The analyses demonstrated CMDQ to be moderately to almost perfectly reliable test of mental health in this specific population over the 14-day interval. The result was supported by a Cronbach’s alpha analysis. Replacing missing data by zero had no significant effect on the result of Kappa.

## Abbreviations

CMDQ: Common mental disorders questionnaire
SCL-SOM: Symptom check list, somatisation subscale
Whiteley-7: A rating scale for illness worry and conviction
SCL-ANX4: Symptom check list, subscale for anxiety
SCL-8: Symptom check list, subscale for mental illness
SCL-DEF6: Symptom Check List, depression subscale
CAGE: A questionnaire for alcohol dependence
SCAN: Schedules for clinical assessment in neuropsychiatry
Dutch COSMIN: Dutch “Consensus-based Standards for the selection of health Measurement Instruments”. www.cosmin.nl
